# Pertuzumab Increases 17-AAG-Induced Degradation of ErbB2, and This Effect Is Further Increased by Combining Pertuzumab with Trastuzumab

**DOI:** 10.3390/ph5070674

**Published:** 2012-06-28

**Authors:** Juliana Bentes Hughes, Marianne Skeie Rødland, Max Hasmann, Inger Helene Madshus, Espen Stang

**Affiliations:** 1 Institute of Pathology, Oslo University Hospital, Rikshospitalet University of Oslo, Oslo 0027, Norway; Email: Juliana.B.Hughes@rr-research.no (J.B.H.); Marianne.S.Rodland@rr-research.no (M.S.R.); Inger.Helene.Madshus@rr-research.no (I.H.M.); 2 Roche Diagnostics GmbH, Pharma Research Penzberg, Nonnenwald 2, Penzberg 82377, Germany; Email: max.hasmann@roche.com (M.H.); 3 Department of Pathology, Oslo University Hospital, Rikshospitalet, Post Box 4950 Nydalen, Oslo 0424, Norway

**Keywords:** 17-AAG, pertuzumab, trastuzumab, Hsp90, ErbB2

## Abstract

ErbB2 is an important oncogenic protein involved in carcinogenesis of, among others, breast, gastric, and ovarian carcinoma. Over-expression of ErbB2 is found in almost 20% of breast cancers, and this results in proliferative and anti-apoptotic signalling. ErbB2 is therefore an important treatment target. Antibodies recognizing full-length ErbB2 are clinically established, and drugs targeting the ErbB2 stabilizing heat shock protein 90 (Hsp90) are under clinical evaluation. We have investigated effects of the ErbB2-binding antibodies trastuzumab and pertuzumab alone and in combination, as well as the effect of the antibodies in combination with the Hsp90 inhibitor 17-AAG. Our results confirm the notion that combination of different ErbB2-binding antibodies more efficiently down-regulates ErbB2 than does one antibody in isolation. Additionally, our data demonstrate that ErbB2 is most efficiently down-regulated upon incubation with anti-ErbB2 antibodies in combination with Hsp90 inhibitors. The combination of anti-ErbB2 antibodies, and especially the combination of antibodies with 17-AAG, did also increase the inhibition of Akt activation of either agent, which could suggest an anti-proliferative effect. In such case, combining these agents could be beneficial in treatment of tumors not responding to trastuzumab only.

## Abbreviations

17-AAG: 17-N-allylamino-17-demethoxygeldanamycin
PAE: Porcine aortic endothelial
EEA1: early endosome antigen 1

## 1. Introduction

ErbB2/HER2 is a member of the Epidermal Growth Factor Receptor (EGFR) family of receptor tyrosine kinases. Eleven ligands have so far been demonstrated to interact with EGFR, ErbB3 and ErbB4 [1,2], but ErbB2 lacks the capacity to bind ligands and is instead activated through heterodimerization with other ErbB proteins [1,3,4]. Importantly, however, ErbB2 is the preferred dimerization partner for the other ErbB proteins [5]. ErbB2 is endocytosis deficient [6,7], and we have previously demonstrated that ErbB2 negatively impacts on EGFR down-regulation [8]. ErbB2 is stabilized by the molecular chaperone heat shock protein 90 (Hsp90) [9,10], and incubation with Hsp90 inhibitors, such as the ansamycin geldanamycin (GA), induces down-regulation of ErbB2 both in cells expressing ErbB2 only and in cells expressing ErbB2 and other ErbB proteins. We have previously demonstrated that GA-induced down-regulation of ErbB2 was more efficient when cells expressed ErbB2 in combination with other ErbB proteins [11,12]. Hsp90 has, in addition to ErbB2, a number of other client proteins, including Akt, that control cell growth [10,13,14], and GA-derivatives such as 17-allylamino-17-demethoxygeldanamycin (17-AAG) are currently in development for cancer treatment [15].

ErbB2 is overexpressed in a number of human cancers, and humanized anti-ErbB2 monoclonal antibodies are important therapeutic agents in several malignant diseases. Trastuzumab is a humanized monoclonal antibody that binds to a membrane-proximal domain of ErbB2 [16]. Trastuzumab has clinical effects, even though its mechanism of action is still not completely understood. Although trastuzumab as single agent has been proved active and well tolerated in treatment of breast cancer with ErbB2 overexpression [17], many tumors overexpressing ErbB2 do not respond to trastuzumab monotherapy, and acquired resistance does often develop [18,19]. *In vitro* studies have, however, demonstrated that a combination of trastuzumab and 17-AAG led to enhanced down-regulation due to lysosomal degradation of ErbB2 in ErbB2-overexpressing breast cancer cell lines [20].

The anti-ErbB2 antibody pertuzumab was demonstrated to bind the dimerization arm of ErbB2 and to thereby block ErbB2 dimerization and downstream tumorigenic signaling [21,22]. We have previously demonstrated that pertuzumab efficiently counteracted EGFR-ErbB2 dimerization and thereby facilitated ligand-induced down-regulation of the EGFR [23]. Recent studies have confirmed beneficial effects of combining trastuzumab and pertuzumab [24,25,26,27,28]. Incubation with antibodies to ErbB2 or to EGFR induces receptor down-regulation with varying efficiency, but combination of antibodies recognizing different epitopes has turned out to be more efficient [29,30,31]. The added effect has been explained both by increased endocytosis due to efficient cross-linking of receptors at the plasma membrane [30], and by inhibition of recycling from endosomal compartments [31]. The combination of trastuzumab and pertuzumab has also been shown to synergistically inhibit cell survival, in part by inhibited activation of Akt [32]. We have in the current work compared the effect of pertuzumab and trastuzumab alone or in combination with or without 17-AAG. Our results support the notion that the combination of pertuzumab, trastuzumab and 17-AAG increases the inhibitory effect on Akt activation of each of the agents, and induces the most efficient down-regulation of ErbB2 from the plasma membrane, leading to lysosomal degradation of ErbB2.

## 2. Experimental

### 2.1. Materials

17-N-Allylamino-17-demethoxygeldanamycin (17-AAG) was purchased from Tocris Bioscience (Bristol, UK). All other chemicals were from Sigma-Aldrich Co. LLC (St. Louis, MO, USA), unless otherwise noted.

### 2.2. Antibodies

Pertuzumab (rhuMAb 2C4) was a gift from Roche (Roche Diagnostics GmbH, Penzberg, Germany). Trastuzumab (Herceptin) was from Roche Pharma AG (Grenzach-Wyhlen, Germany). Mouse anti-ErbB2 (clone TAB250 to the extracellular part), rabbit anti-ErbB2 (clone PAD: Z4881 to the intracellular part), goat anti-mouse IgG-allophycocyanine (APC), and donkey anti-goat IgG-Alexa647 antibodies were from Life Technologies Corporation (San Francisco, CA, USA). Rabbit anti-phospho-Akt (Ser473) was from Cell Signaling Technology (Boston, MA, USA). Goat anti-early endosome antigen 1 (EEA1) (N-19) was from Santa Cruz Biotechnology, Inc. (Santa Cruz, CA, USA). Rabbit anti-β-actin and rabbit anti-tubulin was from Abcam (Cambridge, UK). Donkey anti-mouse IgG-Rhodamine, donkey anti-human IgG-Cy2 and donkey anti-rabbit IgG-peroxidase were from Jackson ImmunoResearch Laboratories, Inc. (West Grove, PA, USA).

### 2.3. Cell Culture and Treatment

Porcine aortic endothelial (PAE) cells stably expressing ErbB2 (PAE.ErbB2) [8] or ErbB2 and ErbB3 (PAE.ErbB2.ErbB3) [12] were grown in Ham’s F-12 (Lonza Group Ltd., Basel, Switzerland) supplemented with 10% vol/vol fetal bovine serum (FBS), and 0.5× Penicillin-streptomycin mixture (Lonza Group Ltd.). The cells were grown in the presence of 30 µg/mL zeocin (Life Technology Incorporation) (PAE.ErbB2) or 30 µg/mL zeocin and 60 µg/mL hygromycin B (Life Technologies Corporation) (PAE.ErbB2.ErbB3). The human cell line SKOv3 was from the American Tissue Culture Collection (ATCC, Manassas, VA, USA) and was grown in DMEM (Lonza Group Ltd.) containing 10% vol/vol FBS (PAA Innovations, Linz, Austria) and 0.5× penicillin-streptomycin mixture. All cell lines were maintained as monolayers at 37 °C in 5% CO_2_.

### 2.4. Immunoblotting

Upon SDS-PAGE, cell lysates were electrotransferred to nitrocellulose membranes (GE Healthcare Life Sciences, Piscataway, NJ, USA). The membranes were incubated with primary and secondary antibodies at 4 °C overnight or at room temperature for 1 h, and proteins were detected using Super Signal West Dura Extended Duration Substrate (Thermo Fisher Scientific, Waltham, MA, USA) and KODAK Image Station 4000R (Carestream, Health, Inc., Rochester, NY, USA).

### 2.5. Degradation of ErbB2

PAE.ErbB2, PAE.ErbB2.ErbB3 and SKOv3 cells were incubated with or without 17-AAG (3 µM), combined with or without pertuzumab (25 µg/mL), trastuzumab (21 µg/mL), or the combination of both antibodies for 5 h at 37 °C in the presence of cycloheximide (25 µg/mL). Upon incubation, cells were lysed, and the lysates were subjected to SDS-PAGE using 10% TGX gels (Bio-Rad, Hercules, CA, USA) for 15 min at 300 V. Immunoblotting and degradation measurements were performed as described [33].

### 2.6. Immunocytochemistry and Confocal Microscopy

PAE.ErbB2 cells were plated on 12 mm coverslips (Menzel-Gläser, Braunschweig, Germany) and incubated in the presence or absence of 17-AAG (3 µM), pertuzumab (25 µg/mL), trastuzumab (21 µg/mL), or the combination of both antibodies and 17-AAG for 1 h at 37 °C. Upon treatment, immunostaining of ErbB2 was performed as described [7]. The cells were mounted with DAKO fluorescent mounting medium and then examined using confocal microscopy (Leica TCS SP, Leica Microsystems AG, Wetzlar, Germany).

### 2.7. Flow Cytometry

Upon incubation as described in figure legends, the cells were trypsinized and resuspended in PBS containing 2% vol/vol FBS and 2 mM EDTA and fixed with 4% PFA for 10 min at room temperature. The fixed cells were incubated with mouse antibody to ErbB2 as described [7]. The anti-ErbB2 antibody at the plasma membrane was detected using APC-conjugated goat anti-mouse IgG antibody and analyzed by flow cytometry using a BD LSR II flow cytometer (BD Biosciences IS, San Jose, CA, USA).

## 3. Results and Discussion

### 3.1. Combination of the Antibodies Pertuzumab and Trastuzumab Induced a Small Cell Type Dependent Down-Regulation of ErbB2

**Figure 1:**
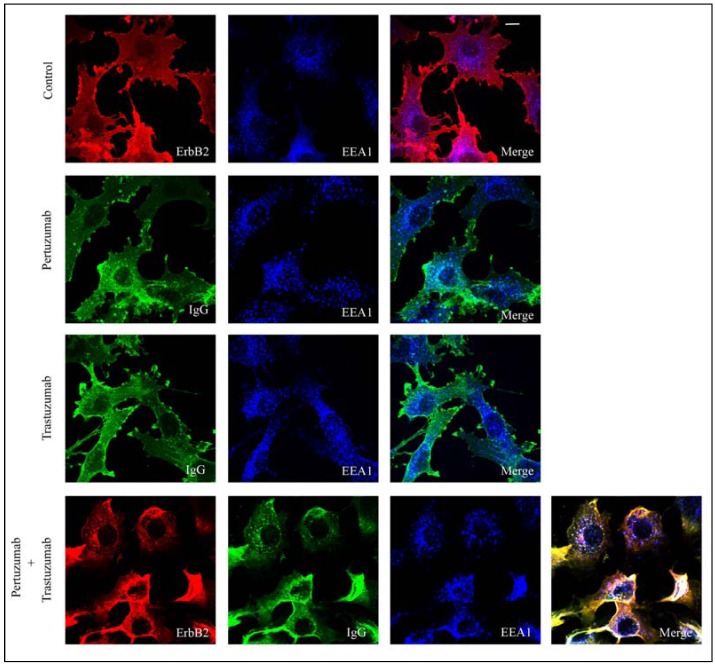
The combination of pertuzumab and trastuzumab enhances antibody-induced endocytosis of ErbB2. PAE.ErbB2 cells were incubated without antibodies (Control), or with pertuzumab only, trastuzumab only, or pertuzumab and trastuzumab in combination for 1 h at 37 °C. The cells were fixed, permeabilized, and immunostained. Cells not incubated with antibodies (Control) were stained using mouse anti-ErbB2, and goat anti-EEA1 antibodies followed by Rhodamine-conjugated donkey anti-mouse, and Alexa647-conjugated donkey anti-goat antibodies. Cells treated with pertuzumab or trastuzumab only were stained using Cy2-conjugated donkey anti-human and goat anti-EEA1 antibodies followed by Alexa647-conjugated donkey anti-goat antibodies. Cells treated with pertuzumab and trastuzumab in combination were stained using Cy2-conjugated donkey anti-human, mouse anti-ErbB2, and goat anti-EEA1 antibodies followed by Rhodamine-conjugated donkey anti-mouse and Alexa647-conjugated donkey anti-goat antibodies. The cells were analyzed by confocal microscopy. Both pertuzumab and trastuzumab are observed in EEA1 positive early endosomes (middle panels, white dots). In cells treated with pertuzumab and trastuzumab, ErbB2 colocalizes with the antibodies in EEA1 positive early endosomes, confirming that ErbB2 was endocytosed (lower panel, white dots). The figure shows one representative experiment out of three independent experiments. Bar, 10 µm.

**Figure 2:**
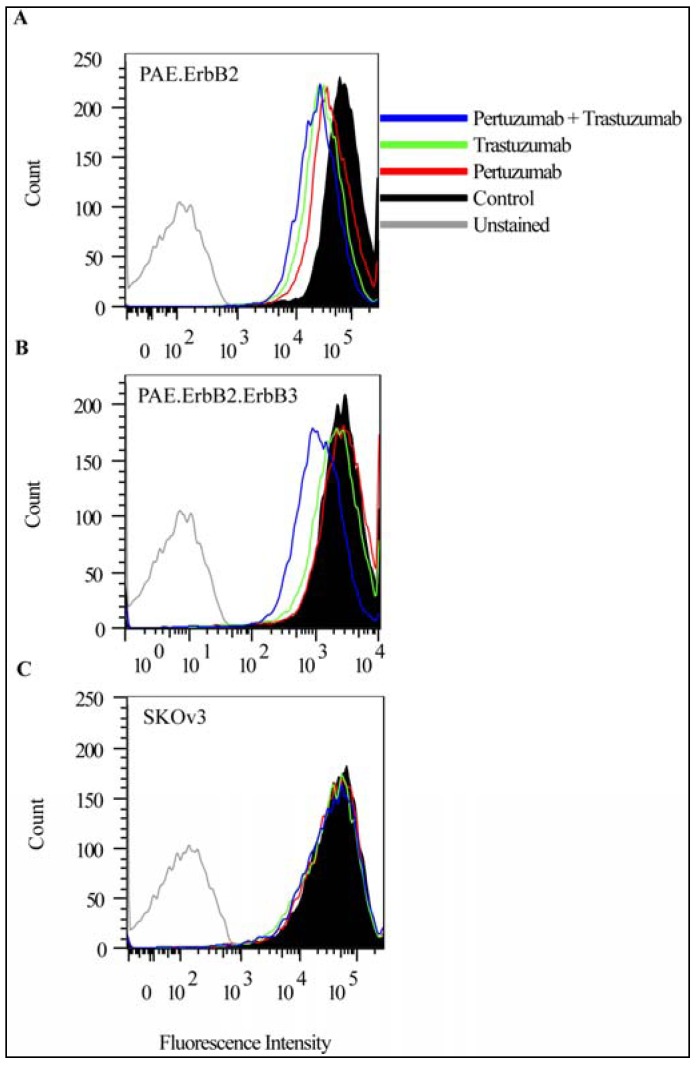
Pertuzumab and trastuzumab have an additive effect on down-regulation of ErbB2 from the plasma membrane in cells expressing ErbB2 and ErbB3 only. PAE.ErbB2 (**A**), PAE.ErbB2.ErbB3 (**B**) and SKOv3 (**C**) cells were incubated without antibodies (Control), or with pertuzumab (25 µg/mL), trastuzumab (21 µg/mL), or pertuzumab and trastuzumab for 24 h at 37 °C. The cells were fixed and immunostained using mouse anti-ErbB2 antibody followed by APC-conjugated goat anti-mouse antibody before the amount of ErbB2 at the plasma membrane was measured by flow cytometry.

Different results have been reported with respect to antibody-induced endocytosis of ErbB2 [30,34]. We therefore initially investigated the effect of trastuzumab or pertuzumab alone and then the effect of the combination of trastuzumab and pertuzumab by confocal microscopy analysis and by flow cytometry. For the confocal microscopy studies, we used PAE cells, which do not express endogenous ErbB proteins, but were stably transfected with ErbB2 (PAE.ErbB2 cells) [8]. The cells were incubated without antibodies, with trastuzumab only, with pertuzumab only or with the combination of both antibodies for 1 h at 37 °C. By confocal microscopy, we then observed partial colocalization of human IgG and early endosome antigen 1 (EEA1) in cells incubated with pertuzumab only and also in cells incubated with trastuzumab only. This suggests that a small fraction of the antibody-ErbB2 complexes was internalized (Figure 1, middle panels). Upon incubation with pertuzumab and trastuzumab in combination, we observed an increase in both the intensity and the number of IgG-positive vesicles (Figure 1, lower panel). Staining for ErbB2 upon fixation, using a mouse anti-ErbB2 antibody, confirmed that ErbB2 was restricted to the plasma membrane in cells not incubated with antibodies (Figure 1, upper panel), but colocalized with pertuzumab and trastuzumab in EEA1 positive endosomes upon incubation with both antibodies (Figure 1, lower panel). To obtain more quantitative results, we measured the amount of ErbB2 at the plasma membrane using flow cytometry, and further to investigate whether the presence of other ErbB proteins affected antibody-induced endocytosis of ErbB2, we in addition to PAE.ErbB2 cells, used PAE cells expressing both ErbB2 and ErbB3 (PAE.ErbB2.ErbB3 cells), and SKOv3 cells. Cells were incubated with trastuzumab or pertuzumab separately or in combination for 24 h at 37 °C. In PAE.ErbB2 cells (Figure 2A) the amount of ErbB2 at the plasma membrane was slightly down-regulated upon treatment with pertuzumab or trastuzumab only, whereas in PAE.ErbB2.ErbB3 cells (Figure 2B), the single antibodies had no significant down-regulatory effect. While the combination of pertuzumab and trastuzumab had minor additive effects on ErbB2 down-regulation in PAE.ErbB2 cells (Figure 2A), it caused a significantly increased down-regulation of ErbB2 in PAE.ErbB2.ErbB3 cells (Figure 2B). In line with our previously reported data [7], our current results demonstrate that trastuzumab did not induce down-regulation of ErbB2 in SKOv3 cells. Also, no effect was observed when these cells were incubated with pertuzumab only or with the combination of pertuzumab and trastuzumab (Figure 2C). The explanation for the different efficiency of down-regulation in either of the two PAE cell lines and the SKOv3 cells is unclear, but could suggest that different cell lines respond differently to incubation with anti-ErbB2 antibodies. Differences in efficiency may be explained by different density of receptors at the cell surface. Based on previous [12] and current flow cytometry data, PAE.ErbB2 cells express roughly 10 times more ErbB2 compared to PAE.ErbB2.ErbB3 cells. Antibody-induced down-regulation of ErbB2 has been explained by antibody-induced clustering of receptors at the plasma membrane, and the increased effect of combining antibodies recognizing different epitopes was explained by the generation of larger antibody-ErbB2 complexes [30]. The extent of antibody-induced cross-linking will most likely depend on receptor density. While incubation with one antibody could possibly induce sufficient cross-linking in PAE.ErbB2 cells expressing a high amount of ErbB2, a combination of different antibodies would probably be required to obtain sufficient cross-linking and endocytosis in cells expressing small amounts of ErbB2, such as PAE.ErbB2.ErbB3 cells. The differences observed when comparing PAE.ErbB2 cells with PAE.ErbB2.ErbB3 cells and SKOv3 cells could also suggest that the expression of other ErbB proteins has effect on antibody-induced down-regulation of ErbB2. Although we have previously demonstrated that pertuzumab inhibits ErbB2 dimerization [23], the extent of antibody induced ErbB cross-linking may be limited by the presence of preformed ErbB2-containing heterodimers. The availability of ErbB2 to added antibodies could also depend on geometry. In fully differentiated cells ErbB proteins are normally expressed at the basolateral side, and this location may be perturbed to varying extent in dedifferentiated cells. Such cell differences could explain some of the previously reported contradictory results with respect to effect of trastuzumab on ErbB2 down-regulation [34].

### 3.2. Both Pertuzumab and Trastuzumab Increase 17-AAG-Induced Down-Regulation of ErbB2

**Figure 3:**
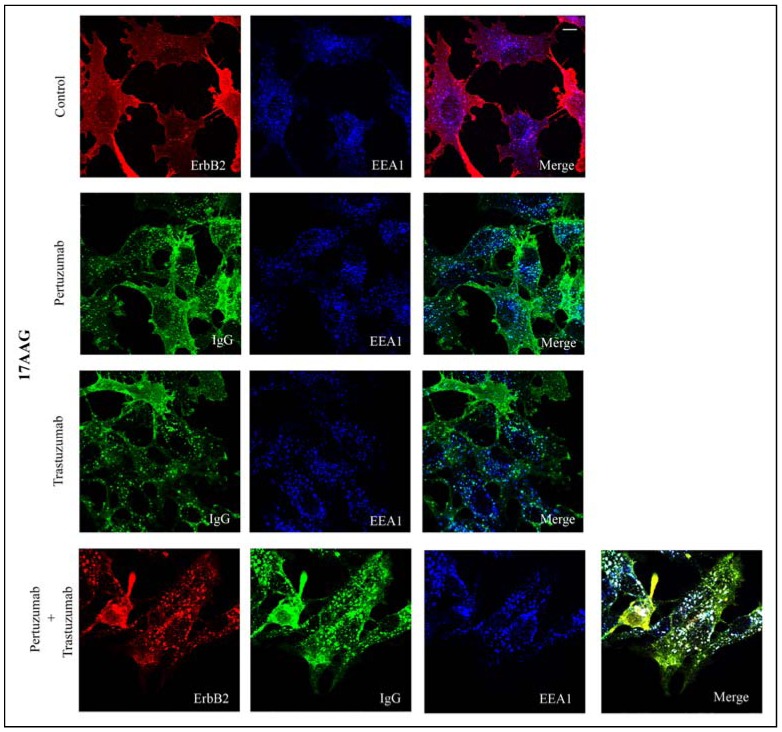
Incubation with either pertuzumab or trastuzumab, or with their combination increases 17-AAG-induced endocytosis of ErbB2. PAE.ErbB2 cells were incubated with 17-AAG (3 µM) only (Control), or with 17-AAG together with either pertuzumab (25 µg/mL), trastuzumab (21 µg/mL), or pertuzumab and trastuzumab in combination for 1 h at 37 °C. Cells treated with 17-AAG only were stained using mouse anti-ErbB2 and goat anti-EEA1 antibodies followed by rhodamine-conjugated donkey anti-mouse and Alexa647-conjugated donkey anti-goat antibodies. Cells incubated with 17-AAG and pertuzumab or with 17-AAG and trastuzumab only were stained using Cy2-conjugated donkey anti-human and goat anti-EEA1 antibodies followed by Alexa647-conjugated donkey anti-goat. Cells incubated with 17-AAG, pertuzumab, and trastuzumab in combination were stained using Cy2-conjugated donkey anti-human, mouse anti-ErbB2, and goat anti-EEA1 antibodies followed by Rhodamine-conjugated donkey anti-mouse and Alexa647-conjugated donkey anti-goat antibodies. The cells were analyzed by confocal microscopy. Endocytosed IgGs were observed to colocalize with EEA1 in early endosomes, and internalized ErbB2 was observed to colocalize with EEA1 in early endosomes (lower panel, white dots). The figure shows one representative experiment out of three independent experiments. Bar, 10 µm.

It is well established that incubation with Hsp90 inhibitors, such as GA and its derivative 17-AAG, induces endocytosis and degradation of ErbB2 [11,12], and previous studies have shown that trastuzumab enhances the effect of 17-AAG on ErbB2 [20,35]. To compare the down-regulatory effect of the antibodies and 17-AAG, PAE.ErbB2 cells were incubated with 3 µM 17-AAG alone, with 17-AAG together with pertuzumab or trastuzumab, or with 17-AAG and both antibodies. Confocal microscopy analysis confirmed that incubation with 17-AAG for 1 h at 37 °C induced endocytosis of ErbB2 (Figure 3, upper panel). When cells were incubated with either of the antibodies in combination with 17-AAG, labeling for human IgG showed a vesicular staining that appeared more intense compared to when cells were incubated with antibodies only (compare middle panels in Figure 3 with middle panels in Figure 1). The number of IgG positive vesicles and the intensity of the vesicular staining did, however, further increase when the cells were incubated with 17-AGG and the combination of pertuzumab and trastuzumab (Figure 3, lower panel).

**Figure 4:**
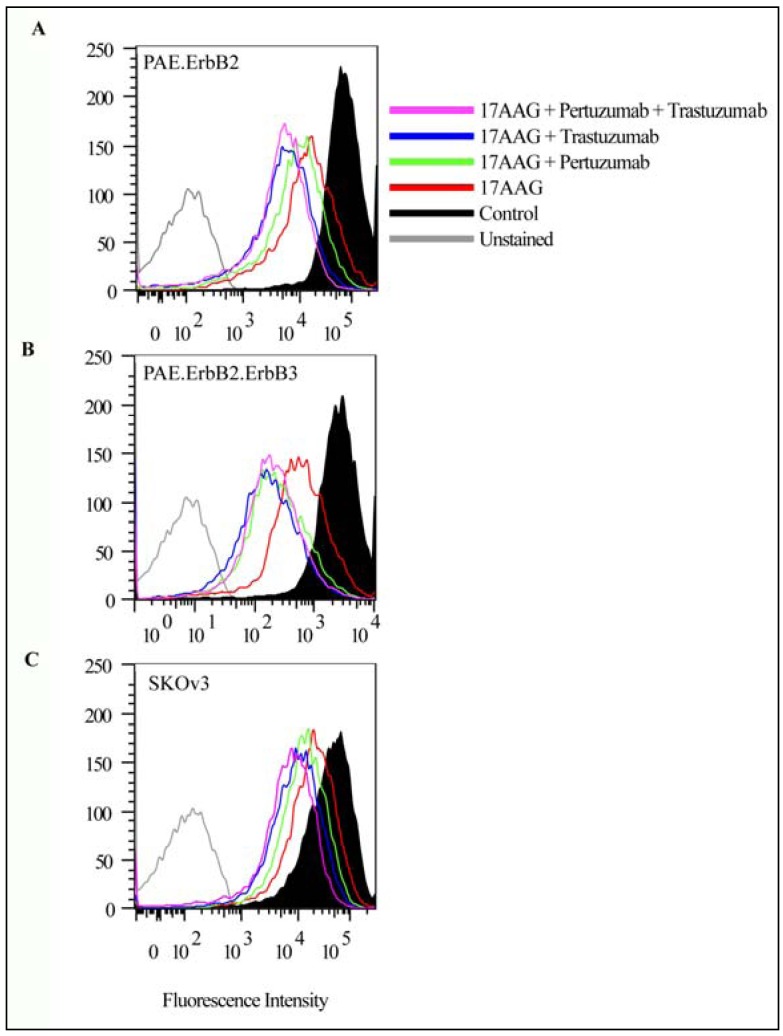
Incubation with pertuzumab and/or trastuzumab potentiates 17-AAG-induced down-regulation of ErbB2 from the plasma membrane. PAE.ErbB2 (**A**), PAE.ErbB2.ErbB3 (**B**) and SKOv3 cells (**C**) were incubated with 17-AAG (3 µM) alone or with 17-AAG and either pertuzumab (25 µg/mL), trastuzumab (21 µg/mL) or pertuzumab and trastuzumab for 24 h at 37 °C. The cells were fixed and immunostained using mouse anti-ErbB2 antibody followed by APC-conjugated goat anti-mouse antibody. The amount of ErbB2 at the plasma membrane was measured using flow cytometry.

Labeling for ErbB2 verified that the incubation with pertuzumab and trastuzumab in combination strongly increased the effect of 17-AAG (compare labeling for ErbB2 in Figure 3 upper and lower panels). These results suggest (as previously demonstrated for trastuzumab) [20,35], that also pertuzumab increased the effect of 17-AAG and that a combined incubation with pertuzumab and trastuzumab has an additive effect. To compare the effects quantitatively, and to compare the effect in different cell lines, we used flow cytometry analysis. PAE.ErbB2, PAE.ErbB2.ErbB3, and SKOv3 cells were incubated with either pertuzumab or trastuzumab or with both antibodies in the presence of 3 µM 17-AAG for 24 h at 37 °C. As demonstrated in Figure 4, the amount of ErbB2 was clearly down-regulated upon incubation with 17-AAG for 24 h, and in line with our previous data [12], the down-regulation of ErbB2 appeared to be stronger in PAE.ErbB2.ErbB3 cells (Figure 4B) compared to in PAE.ErbB2 cells (Figure 4A).

Both pertuzumab and trastuzumab enhanced the effect of 17-AAG in all cell lines tested. In PAE.ErbB2.ErbB3 and in SKOv3 cells, the two antibodies showed comparable effects, while in PAE.ErbB2 cells pertuzumab had a slightly reduced effect compared to trastuzumab. The combination of pertuzumab and trastuzumab together with 17-AAG appeared to give the most efficient down-regulation even though the effect of adding pertuzumab in addition to trastuzumab seemed limited.

### 3.3. Pertuzumab and Trastuzumab Potentiate 17-AAG-Induced Degradation of ErbB2

**Figure 5:**
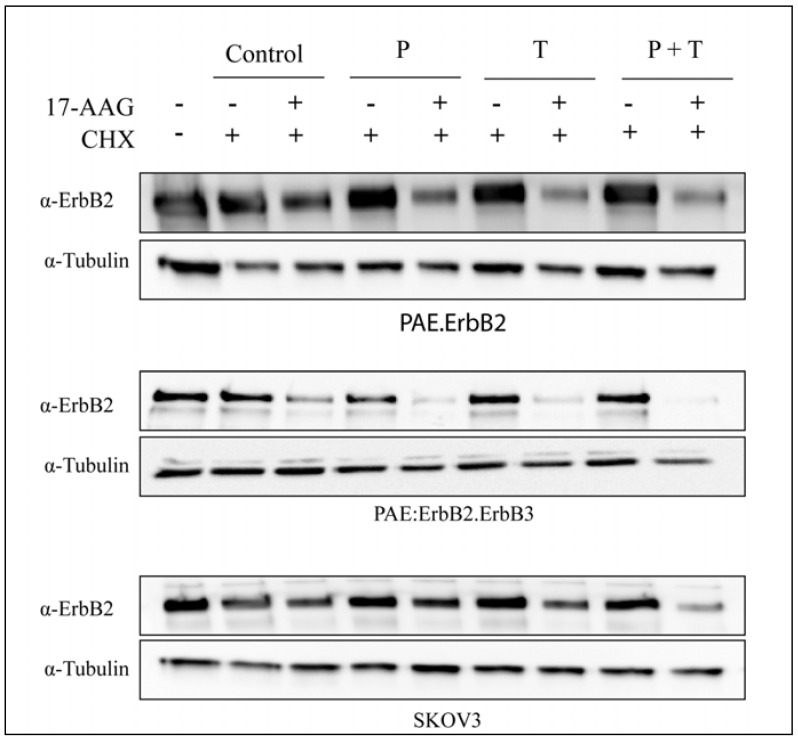
Incubation with pertuzumab and trastuzumab increases 17-AAG-induced degradation of ErbB2. PAE.ErbB2, PAE.ErbB2.ErbB3 and SKOv3 cells were incubated with pertuzumab (25 µg/mL), trastuzumab (21 µg/mL) and their combination in the presence or absence of 17-AAG (3 µM) for 5 h at 37 °C in the presence of cycloheximide (CHX; 25 µg/mL). The cells were lysed and subjected to SDS-PAGE and immunoblotting with rabbit anti-ErbB2 antibody. Immunoblotting for Tubulin was used as loading control. The combination of pertuzumab, trastuzumab and 17-AAG strongly increased 17-AAG-induced degradation of ErbB2 in all cell lines tested.

**Figure 6:**
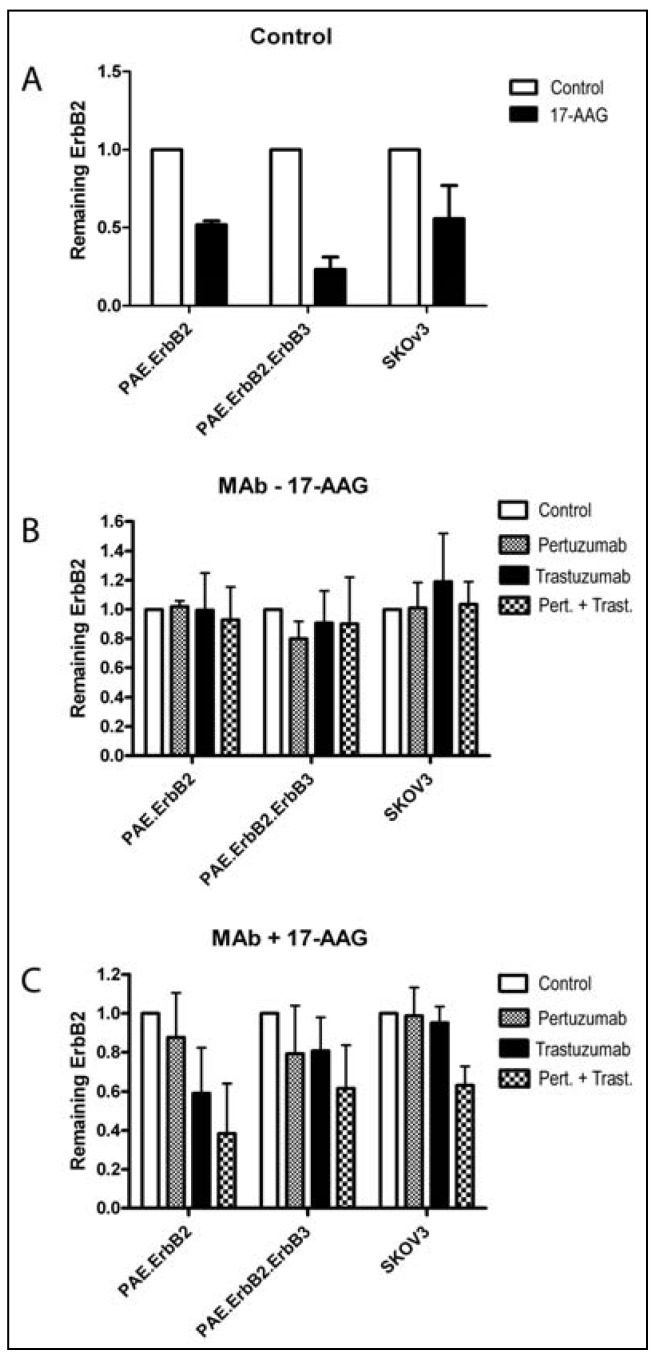
Quantification of pertuzumab-, trastuzumab-, and/or 17-AAG-induced degradation of ErbB2. The intensity of the bands in the Western blots demonstrated in Figure 5 was measured, and for each condition the intensity for ErbB2 was normalized to tubulin. The calculated values thus reflects the amount of ErbB2 remaining in cells after the various treatments. (**A**) Effect of incubation with 17-AAG. The relative intensity of ErbB2 in cells treated with CHX only was set to 1; (**B**) Effect of incubation with pertuzumab and/or trastuzumab. The relative intensity of ErbB2 in cells treated with CHX only was set to 1; (**C**) Effect of incubation with antibodies and 17-AAG in combination. The relative intensity of ErbB2 in cells treated with 17-AAG and CHX, but no antibodies, was set to 1, and the control values do thus correspond to the respective 17-AAG values in (**A**).

The confocal microscopy and flow cytometry data demonstrated that incubation with pertuzumab and trastuzumab in combination induced down-regulation of ErbB2 from the plasma membrane more efficiently than did either antibody alone. Also, addition of the antibodies increased the 17-AAG-induced down-regulation of ErbB2. Given the probability that ErbB2 can signal also when localized to early endosomes, it is important to know under which conditions ErbB2 is sorted to late endosomes and lysosomes and degraded. Incubation with 17-AAG normally results in lysosomal degradation of ErbB2. To investigate to what extent endocytosed ErbB2 was efficiently degraded, PAE.ErbB2, PAE.ErbB2.ErbB3 and SKOv3 cells were incubated with each of the two antibodies alone or in combination in the presence or absence of 17-AAG for 5 h at 37 °C in the presence of cycloheximide. Incubation with 17-AAG alone induced degradation of ErbB2 in all cell lines tested (Figure 5 and Figure 6A). 17-AAG induced degradation of ErbB2 was most prominent in PAE.ErbB2.ErbB3 cells. This is in line with our previous results, demonstrating that expression of ErbB3 increases the rate of GA-induced down-regulation of ErbB2 [12]. Incubation with antibodies alone did not induce detectable degradation of ErbB2 in any of the cell lines even when the two antibodies were combined (Figure 5 and Figure 6B). However, when PAE.ErbB2 and PAE.ErbB2.ErbB3 cells were treated with either of the antibodies in combination with 17-AAG, a substantial amount of ErbB2 was degraded already upon incubation for 5 h. In PAE.ErbB2 cells, this effect was strongest upon incubation with trastuzumab and 17-AAG (Figure 5 and Figure 6C). However, importantly, in both cell lines, the combination of pertuzumab and trastuzumab together with 17-AAG gave the most pronounced effect. In SKOv3 cells, neither trastuzumab, nor pertuzumab alone increased 17-AAG-induced degradation. However, also in these cells did the combined incubation with trastuzumab and pertuzumab strongly increase 17-AAG induced degradation of ErbB2 (Figure 5 and Figure 6C). It has previously been shown that the combination of trastuzumab and 17-AAG induced enhanced ubiquitination, endocytosis and lysosomal degradation of ErbB2 [20], and it is possible that pertuzumab has the same effect when combined with 17-AAG. The strong effect on degradation of ErbB2 when combining pertuzumab, trastuzumab and 17-AAG in all the cell lines examined was most likely induced by complementary mechanisms including efficient endocytosis due to the formation of large antibody-ErbB2 complexes [29,30] and increased 17-AAG induced ubiquitination [20], as well as inhibition of recycling due to antibody-mediated receptor cross-linking [31] and ubiquitin-mediated lysosomal sorting of ErbB2 [20].

### 3.4. 17-AAG Enhances the Inhibitory Effect on Akt Activation Induced by the Combination of Pertuzumab and Trastuzumab

In addition to endocytosis and degradation of ErbB2, inhibition of cell growth is an important effect of both anti-ErbB2 antibodies and Hsp90-inhibitors. The combination of trastuzumab and pertuzumab has previously been shown to synergistically inhibit activation of Akt [32]. Akt is itself an Hsp90 client and will upon incubation with Hsp90-inhibitors over time be degraded [13]. Incubation with GA has, however, in addition also been demonstrated to induce a rapid decrease in Akt activity due to increased dephosphorylation of Akt prior to degradation of Akt itself [36]. To investigate whether the combination of the antibodies with 17-AAG had an additive inhibitory effect on Akt activation, we therefore incubated cells with each of the two antibodies alone and also in combination with or without 17-AAG for 1 h at 37 °C. In PAE.ErbB2 cells and SKOv3 cells we observed no significant effects on Akt activity (data not shown).

**Figure 7:**
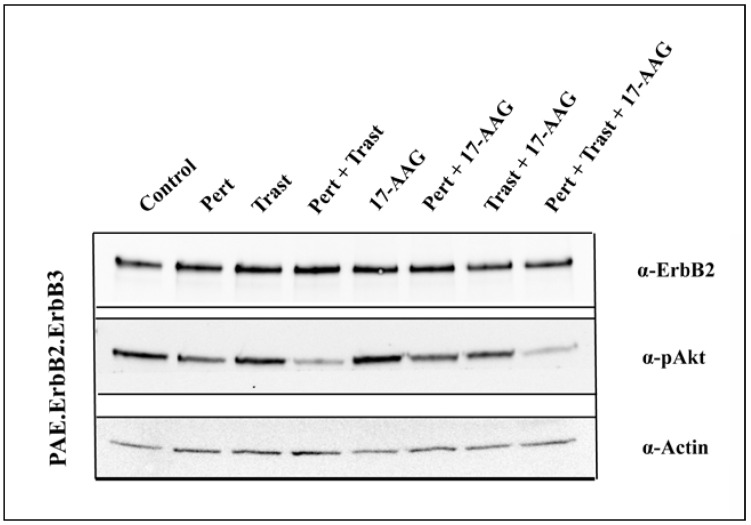
Incubation with 17-AAG increases pertuzumab- and trastuzumab-induced inhibition of Akt activation. PAE.ErbB2.ErbB3 cells were incubated with pertuzumab (25 µg/mL), trastuzumab (21 µg/mL) and their combination in the presence or absence of 17-AAG (3 µM) for 1 h at 37 °C. The cells were lysed and subjected to SDS-PAGE and immunoblotting with rabbit anti-ErbB2 and rabbit anti-phospho-Akt (Ser473) antibodies. Immunoblotting for actin was used as loading control. The combination of pertuzumab and trastuzumab induced a strong decrease in Akt activation, and this effect was further increased when 17-AAG was added along with the antibody combination.

On its own, each agent had minimal effects also in PAE.ErbB2.ErbB3 cells. However, in these cells the combination of pertuzumab and trastuzumab induced a strong decrease in Akt phosphorylation, and this effect was further increased when 17-AAG was added along with the combined antibodies (Figure 7). These results confirm the beneficial effect of combining several different agents, but also emphasize important cell line dependent differences. ErbB3, which depends on dimerization with the EGFR or with ErbB2 for efficient activation, is due to its six docking sites for phosphatidylinositol 3-kinase, the most potent Akt activator of the ErbB proteins (reviewed in [37]). The PAE.ErbB2 cells do not express ErbB3, and also the SKOv3 cells used in the current study lack or minimally express ErbB3 [7]. The strong effects of the combination of pertuzumab and trastuzumab in PAE.ErbB2.ErbB3 cells can thus probably be explained by antibody-induced inhibition of ErbB2-ErbB3 dimerization [32], and the further increase upon incubation with 17-AAG may be caused by 17-AAG-induced internalization of ErbB2 and thus decreased amounts of ErbB2 available for dimerization with ErbB3.

## 4. Conclusions

Altogether, our results show that the combination of antibodies recognizing different regions (and thereby different epitopes) on ErbB2 in combination with 17-AAG gives the most efficient plasma membrane down-regulation and degradation of ErbB2 *in vitro*. Such combination does also give the most efficient decrease in Akt activity, which might suggest an anti-proliferative effect. Since not all ErbB2-overexpressing tumors respond to trastuzumab monotherapy, and since acquired resistance frequently develops, the combinations of these agents may prove therapeutically beneficial. It should however, be noted that the observed effects varied between the different cells used. These differences probably depend on the amount of ErbB2 expressed, and also on expression of other members of the ErbB family, especially ErbB3. Altogether, this emphasizes the importance of thorough molecular characterization of the cancer cells to tailor and optimize treatment.
